# Automated Diagnosis of COVID-19 Using Deep Features and Parameter Free BAT Optimization

**DOI:** 10.1109/JTEHM.2021.3077142

**Published:** 2021-05-03

**Authors:** Taranjit Kaur, Tapan K. Gandhi, Bijaya K. Panigrahi

**Affiliations:** Department of Electrical EngineeringIndian Institute of Technology Delhi (IIT Delhi)New Delhi110016India

**Keywords:** COVID-19, diagnosis, deep features, parameter free BAT optimization

## Abstract

**Background:** Accurate and fast diagnosis of COVID-19 is very important to manage the medical conditions of affected persons. The task is challenging owing to shortage and ineffectiveness of clinical testing kits. However, the existing problems can be improved by employing computational intelligent techniques on radiological images like CT-Scans (Computed Tomography) of lungs. Extensive research has been reported using deep learning models to diagnose the severity of COVID-19 from CT images. This has undoubtedly minimized the manual involvement in abnormality identification but reported detection accuracy is limited. **Methods:** The present work proposes an expert model based on deep features and Parameter Free BAT (PF-BAT) optimized Fuzzy K-nearest neighbor (PF-FKNN) classifier to diagnose novel coronavirus. In this proposed model, features are extracted from the fully connected layer of transfer learned MobileNetv2 followed by FKNN training. The hyperparameters of FKNN are fine-tuned using PF-BAT. **Results:** The experimental results on the benchmark COVID CT scan data reveal that the proposed algorithm attains a validation accuracy of 99.38% which is better than the existing state-of-the-art methods proposed in past. **Conclusion:** The proposed model will help in timely and accurate identification of the coronavirus at the various phases. Such kind of rapid diagnosis will assist clinicians to manage the healthcare condition of patients well and will help in speedy recovery from the diseases. ***Clinical and Translational Impact Statement***— The proposed automated system can provide accurate and fast detection of COVID-19 signature from lung radiographs. Also, the usage of lighter MobileNetv2 architecture makes it practical for deployment in real-time.

## I. Introduction

An outbreak of coronavirus infection (SARS-CoV-2) emerged in December 2019, and by the beginning of the year 2020, the World Health Organization (WHO) announced it as a global pandemic [1]–[3]. Globally, the confirmed coronavirus cases have reached 166 million by 22, May 2021 [4] and are continuously on an increase (https://www.worldometers.info/coronavirus/). The medical experts and the researchers are working together for a better understanding of COVID-19 etiology. Novel strategies are being underway that can better control its spread. The conventional testing procedure is based on reverse transcription-polymerase chain reaction (RT-PCR) and nucleic acid sequencing from the virus. Although, RT-PCR being the gold standard, the procedure is time-consuming, needs to be re-iterated, and has considerable false-negative results. In such scenarios, CT-Scans of affected person plays an important role in better management of health condition. The variations like ground-glass opacities and pulmonary consolidation in CT images are an important biomarker for COVID-19 detection which can help in prompt identification of suspicious cases thereby saving crucial time and readily isolating the patient [5], [6]. Also, the wide accessibility of CT scanners makes this task quicker. Adding to this, Machine learning (ML) and Deep learning (DL) methods are evolving rapidly that can lessen the workload of the medical experts by providing an automatic interpretation from a huge data sets [7]–[10], [39]. Zhao *et al* [11] developed a transfer learned DenseNet model for the classification of CT scan images into COVID + *ve* and Normal categories. The researchers attained an accuracy value of 84.7% and an F1 score of 85.3% on a database encompassing 195 Normal and 275 COVID + *ve* scans. Kaur and Gandhi [12] introduced a transfer learning-based approach for COVID-19 diagnosis using ResNet50 and MobileNetv2. The system was validated on 250 COVID + *ve* and 246 normal scans. The authors attained an accuracy of 98.35%. Loey *et al.* [13] investigated five different transfer learned (DTL) models, i.e., GoogleNet, AlexNet, VGGNet16, VGGNet19, and ResNet50 for COVID-19 classification using the database provided by [11]. The researchers pooled these learned mathematical models with augmentation and Conditional Generative Adversarial Networks (CGAN). The authors established that ResNet 50 is the best, resulting in a test classification accuracy of 82.91%. Soares *et al.* [14] proposed the eXplainable Deep Learning approach (xDNN) to classify if the subject is infected by SAR-Cov-2 or not. They achieved an F-score value of 97.31% with an accuracy value of 97.38%. Pathak *et al* [15] proposed a deep bidirectional long short-term memory network with a Mixture Density model (DBM) for classification of CT scans as COVID + *ve* and normal. The hyperparameters of the DBM model were tuned using the Memetic Adaptive Differential Evolution (MADE)algorithm. The authors attained an accuracy of 98.37% and an F1-score of 98.14% under the train test ratios of 60: 40 over a dataset having 1252 + *ve* and 1230 − *ve* COVID scans. Surveying the literature reveals that the usage of the CT scans in diagnosing COVID-19 is drawing attention due to the inefficiency of the medical testing kits. Also manually inspecting each CT image in the entire volume is tiresome and challenging for the medical experts. As the number of cases are increasing by multi-folds daily, automatic Computer-aided diagnosis (CAD) systems is the need of the hour. Although CAD based on ML and DL are being developed and that have aided in speedy detection of the virus, however, not all the reported works are reproducible as the employed datasets are not available for public usage. Also, the DL requires large annotated training datasets for good detection results. To address this limitation, several works have employed transfer learned models on the benchmark dataset provided by Soares *et al.* [14] but the diagnosis accuracy is limited [12], [14], [15].Encouraged by the benefit of the employing transfer learning on a small pathological database and to improvise the classification results over existing methods, the present work proposes a system based on the integration of MobineNetv2 architecture with Parameter Free BAT(PF-BAT) optimized Fuzzy KNN(FKNN) classifier for the automated classification of COVID-19 CT scans. Typically, the deep characteristic features are extracted from the fully connected (‘*new_fc’*) layer of the transfer learned MobileNetv2 which are then fed to the FKNN whose hyper-parameters are fine-tuned via the PF-BAT. Summarizing the key contributions are as follows:

• A novel diagnosis model is proposed based on the integration of deep features and one of the recent metaheuristic optimization algorithms.
• The proposed model overcomes the drawback of the manual hyperparameter tuning via employing the PF-BAT optimization algorithm.
• The proposed model attains an accuracy of 99.38% on the COVID-19 CT scan dataset that is better than the existing state of the art models.

## II. Material and Methods

### A. COVID CT Database Description

**FIGURE 1.**
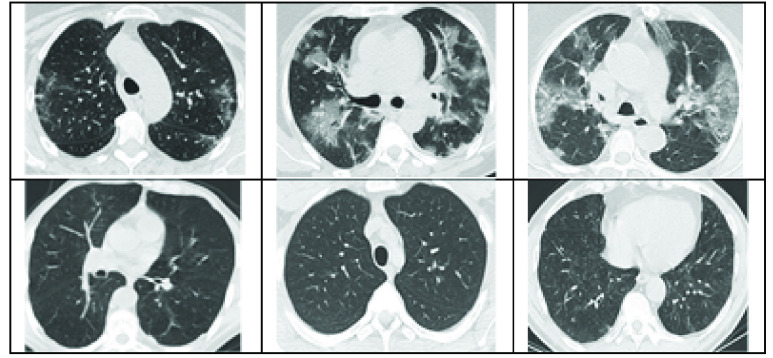
Example of + *ve* (upper row) and − *ve* (lower-row) COVID scans from the dataset [14].

In this proposed work, we have considered the SARS-CoV-2 CT image database provided by Soares *et al.* [14]. The database consists of 2D CT images of 60 (28 females and 32 males) SARS-CoV-2 infected patients and images from 60 control subjects. Therefore, in total 2482 images are made available with approx. 1252 + *ve* and 1230 − *ve* for the virus [14]. The train-validation splits given in Table 1, are as per the base paper without an indication whether they were subject independent or not. Example + *ve* and − *ve* COVID scans from the dataset are shown in Fig. 1.

**TABLE 1:** Data Split Information

Data Set	Non-COVID	COVID	Total
Train	983	1003	1986
Validation	246	250	496

### B. Methodology

This section provides the mathematical background for the blocks used in the proposed system.

#### 1) MobileNetv2

**FIGURE 2.**
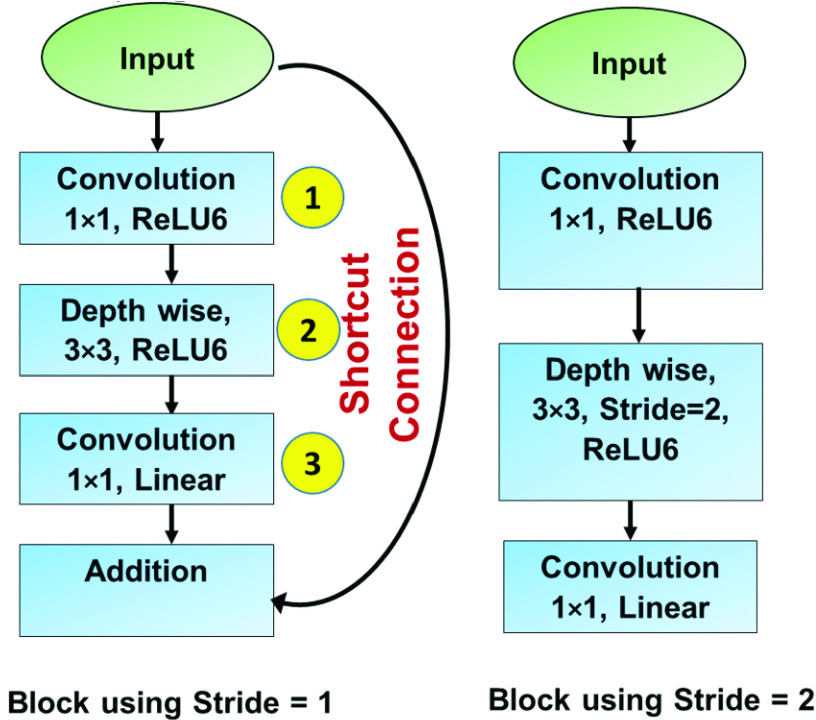
Building blocks of MobileNetv2 [16].

The structural design of MobileNetv2 is motivated by the MobileNetv1 [16]. It uses depth-wise separable convolutions as the building blocks. In comparison to the V1, it has two new additions: a) depthwise separable convolution, and b) inverted residuals [16]. The MobileNetv2 model is pretrained on ImageNet dataset with 1.4 million images and 1000 classes. The basic building block is shown below as Fig. 2. Input, output dimensions at the different stages of the structure given in Fig. 2. is mathematically represented in Table 2.

**TABLE 2:** Input and Output at Different Stages of Bottleneck Residual Block

Stage	Input	Output
1	$u\times v\times k$	$u\times v\times (tk)$
2	$u\times v\times tk$	$\frac {u}{s}\times \frac {v}{s}\times (tk)$
3	$\frac {u}{s}\times \frac {v}{s}\times tk$	$\frac {u}{s}\times \frac {v}{s}\times k'$

$u\times v$: Block size; $t$: Expansion factor; $s$: stride; $k,k'$: Input, output channels

The general architecture of the model is summarized in Table 3 beginning with convolutional layer (having 32 filters) and subsequently followed by residual bottleneck layers which are 19 in number [16].

**TABLE 3:** General Architecture of Mobilnetv2

Input ($\text{m}^{2}\times \text{p}$)	Operator	Expansion	Stride	Repetitions	Channels
m=224; p=3	C	–	2	1	32
m=112; p=32	B	1	1	1	16
m=112; p=16	B	6	2	2	24
m=56; p=24	B	6	2	3	32
m=28; p=32	B	6	2	4	64
m=14; p=64	B	6	1	3	96
m=14; p=96	B	6	2	3	160
m=7; p=160	B	6	1	1	320
m=7; p=320	C $1\times 1$	–	1	1	1280
m=7; p=1280	avgpool $7\times 7$	–	–	1	–
m=1; p=1280	C $1\times 1$	–		–	$k$

C: Conv2d; B: Bottleneck; $k$: Kernel Size

Depthwise separable convolution replaces conventional convolution via two processes. The first process works by applying a different convolution to every feature map which is known as feature map-wise convolution. The resultant feature maps are stacked together to be treated by the second process that is pointwise convolution. In the second process, all the feature maps undergo convolution using a kernel of size $1\times 1$. Differing from the conventional convolution that involves the processing of the image across height, width, and channel dimensions simultaneously, the depthwise convolution processes the image via height and width dimensions in the first phase and via channel during the second phase. The computational cost of the convention convolution process($C_{Conven}$) and depth-wise separable convolution($C_{Depth-seperable}$) is mathematically given as:

\begin{align*} C_{Conven}=&h_{p} w_{p} d_{p} d_{q} k^{2} \tag{1}\\ C_{Depth-seperable}=&h_{p} w_{p} d_{p} (d_{q} +k^{2}) \tag{2}\\ \frac {C_{Conven}}{C_{Depth-seperable}}=&\frac {d_{q} \times k^{2}}{d_{q} +k^{2}}\tag{3}\end{align*}

In the above equation $p$ and $q$ are input/output layer index, ($h_{\mathrm {p}}$, $w_{\mathrm {p}}$, $d_{\mathrm {p}}$) are the height, width, and the count of input feature maps. Also $k$, $d_{\mathrm {q}}$ denotes the kernel size and number of the output feature maps respectively. It is the lesser number of the residual connections between the first and the last feature maps that make MobileNetv2 memory efficient. MobileNetv2 is selected in the present paper as this model is faster than other deep models for the similar level of detection accuracy [16]. In comparison to V1 structure, it employs two times fewer operations, and 30% lesser parameters [16], [17]. This enables its storage and implementation easier on a simple computing platform making it useful for applications in real-time [12], [16].

#### 2) Fuzzy KNN(FKNN) Classifier

FKNN classifier improves upon the conventional KNN classifier by adding the concept of fuzzy logic to the KNN. FKNN algorithm operates by allocating the membership as a mathematical relation of the exemplar distance vector from its $k$-neighbors and the corresponding neighbor’s memberships in possible classes. For an exemplar vector, the fuzzy memberships corresponding to different classes are assigned according to the following formula [18]:

\begin{equation*} mb_{i} (x)=\frac {\sum \limits _{j=1}^{k} {mb_{ij} \left ({{\frac {1}{\vert \vert x-x_{j} \vert \vert ^{\frac {2}{b-1}}}} }\right)}}{\sum \limits _{j=1}^{k} {\left ({{\frac {1}{\vert \vert x-x_{j} \vert \vert ^{\frac {2}{b-1}}}} }\right)}}\tag{4}\end{equation*}

where $i=1,2\ldots \ldots,C$ and $j=1,2,\ldots \ldots,k$. Also, $C$ denotes the class count & $k$ as the nearest neighbor number, $b$ specifies the fuzzy strength metric, $\vert \vert x-x_{j} \vert \vert $ as the distance (Euclidean) measure between an exemplar $x$ and its $j^{\mathrm {th}}$ closest neighbor, and $mb_{ij} $ specifies the degree of membership of exemplar $x_{j} $ from the training pool to the $i^{th}$ class. Various methods exist for defining $mb_{ij} $ and the popular among them are crisp membership and constrained fuzzy membership.

\begin{align*} mb_{ij} (x)=\left \{{{\begin{array}{lll} \eta +\left ({{\displaystyle \frac {p_{j}}{K}} }\right)\times \left ({{1-\eta } }\right),&\quad if \, j=i \\[3mm] \left ({{\displaystyle \frac {p_{j}}{K}} }\right)\times \left ({{1-\eta } }\right),&\quad if \,  j\ne i \\ \end{array}} }\right \}\tag{5}\end{align*}

where $p_{j} $ is the number of the exemplars that belong to the class $j$, in the $K$ nearest training exemplars of $x$, and $\eta \in [{0,1}]$ signifies the bias parameter.

Also, for binary class scenario $mb_{ij} $ must satisfy the following eq. (6) [18]

\begin{equation*} \sum \limits _{i=1}^{2} {mb_{ij}} =1,\quad j=1,2,\ldots \ldots,n\tag{6}\end{equation*}

where

\begin{equation*} mb_{ij} \in [0,\,\,1]\end{equation*}

Depending upon the value of $mb_{ij} $ for every class under consideration, a test sample or exemplar is allocated to appropriate class to which it exhibits the maximum membership, i.e.,

\begin{equation*} c(x)=\arg [\max (mb_{i} (x))]_{i=1}^{2}\tag{7}\end{equation*}

The motivation behind using FKNN is its excellent performance over a wide variety of disease diagnosis problems like Thyroid [19], Parkinson [20], [21] and seizure [22].

#### 3) Parameter Free BAT (PF-BAT) Optimization

PF-BAT is an improvement over the BAT algorithm developed by Yang [23]. The conventional BAT algorithm follows certain updation rules that guide them on their foraging behavior as specified below [23]:

\begin{align*} freq_{j}=&freq_{\min } +(freq_{\max } -freq_{\min })\delta \tag{8}\\ \,v_{j}^{t}=&v_{j}^{t-1}+(x_{j}^{t-1} -x\ast)freq_{j} \tag{9}\\ x_{j}^{t}=&x_{j}^{t-1} +v_{j}^{t} \tag{10}\\ x_{new}=&x_{old} +\varepsilon B^{t} \tag{11}\\ B_{j}^{t+1}=&\alpha B_{j}^{t} \tag{12}\\ r_{j}^{t+1}=&r_{j}^{0} (1-\exp (-\mu t))\tag{13}\end{align*}

where $x_{j} $ is position, $v_{j} $ is velocity, $t$ is the time step, $freq_{j} $ is the pulse frequency, $B_{j} $ is the loudness, and $r_{j} $ is the pulse emission rate (corresponding to $j^{\mathrm {th}}$ BAT). $\delta $ and $\varepsilon $ denotes the random numbers in the range [0, 1] and [−1, 1] respectively. Also, $\alpha $ and $\mu $ are fixed constants.

The exploration and exploitation abilities of the BAT are impecunious [24]. To alleviate this constraint, the variation structure was introduced, motivated by the works in [25], [26]. The new position update mechanism is given below [27]:

\begin{align*} x_{j}^{t} =\left({1-\frac {x\ast }{x_{j}^{t-1}}}\right)\times freq_{j} \times x\ast +\left({\frac {x\ast }{x_{j}^{t-1}}}\right)\times freq_{j} \times pbest_{j} \\\tag{14}\end{align*}

Here, ${pbest}_{j}$ is the best solution previously identified by each bat and $x\ast $ is the global best solution. As the improvised method removes the velocity update mechanism, so it is called as PFree BAT(PF-BAT). The proposed PF-BAT differs from the existing works by Fister *et al.* [28] as it eliminates the velocity update equation and doesnot involve any extensive experimental evaluation in determining the appropriate value of the five control parameters. Improved performance of the PF-BAT on standard benchmark mathematical functions and a variety of medical image classification tasks has been already demonstrated in the previous work [27]. Also, the superiority of PF-BAT enhanced FKNN over other machine learning algorithms over a broad variety of classification problems has already been established in our previous works [29]. In the present work, its performance has been investigated over COVID CT scan image dataset.

#### 4) Proposed Methodology

**FIGURE 3.**
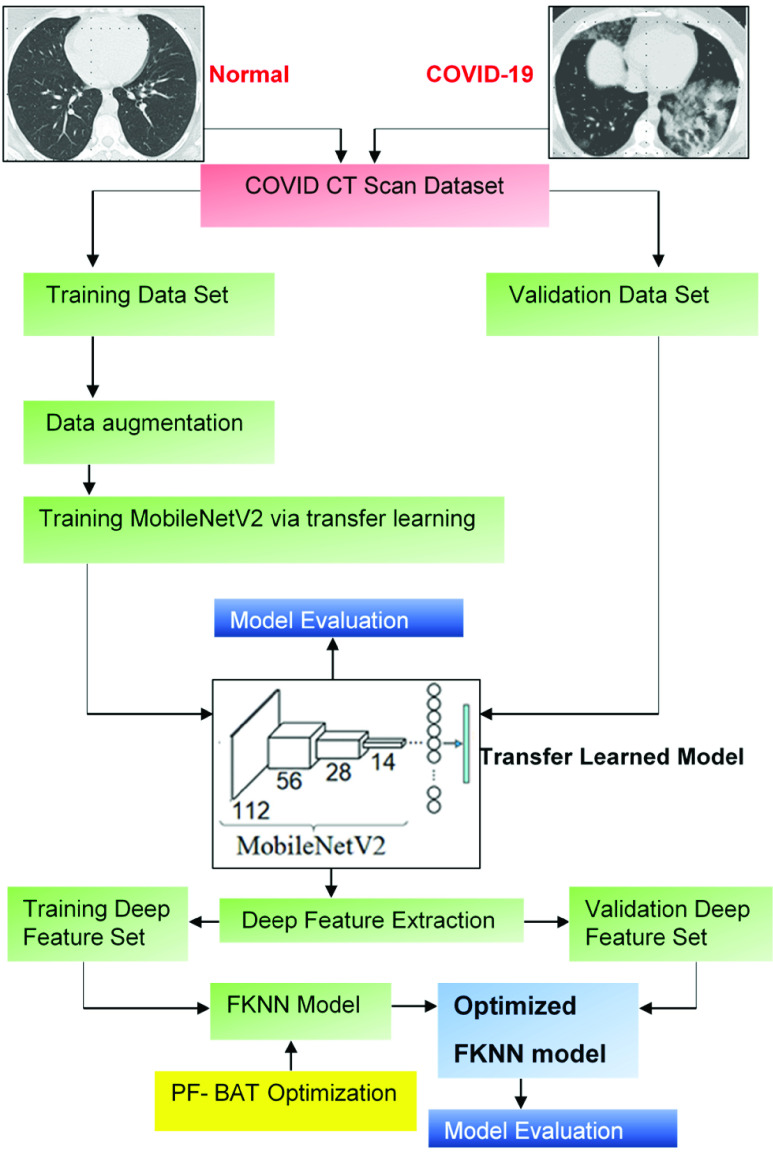
Proposed methodology.

In the present sub-section, the hyper-parameter tuning issue of the FKNN classifier is overcome via using the PF-BAT optimization algorithm as manual tuning is a time-intensive process. The proposed methodology is shown in Fig. 3.

As illustrated, the designed methodology is separated into four phases; 1) data preparation, 2) transfer learning, 3) feature extraction, and 4) classification. In the data preparation part, the database is distributed in 8:2 proportion (i.e., 80% of the whole images are employed for training and rest for validation). For better generalization and prevention of overfitting, data augmentation is employed over training scan images. Augmentation involves the following processes, i.e., random translation in pixel range [−3, 3], random shear in the range from [−0.05, 0.05], and random rotation in the range [−10, 10].

In the second phase, a pre-trained MobileNetv2 [17] model is used which is fine-tuned on the CT scan images through the concept of transfer learning. It is evident that the first few layers of the pre-trained model comprise only edge and color-associated information, whereas more specific features are present in later layers. Hence, parameters of the initial layers require very less or no fine-tuning [12], [30]. Based on these observations, we have fine-tuned the last three layers of MobileNetv2 by replacing them with a fully connected (‘*fc*’) layer, a softmax layer, and a classification output layer. The size of the ‘*fc*’ layer is equal to the number of classes (i.e., 2) in the new classification task [31], [32]. Arithmetically: Let *Model* = { *MobileNetv2*} be the pre-trained architecture. Let ($X_{inpu\mathrm {t}}$, $Y_{output}$) be the present CT scan image database; having ‘$N$’ images with the set of labels as $Y_{output}= \{y\vert y \epsilon \{{\it Non-COVID}; {\textit{COVID}}\}\}$. The training and validation pools are represented as ($X_{train}$, $Y_{train}$) and ($X_{val}$, $Y_{val}$). The training data is further allocated into mini-batches ($n$), such that ($X_{i}$, $Y_{i}) \epsilon $ ($X_{train}$, $Y_{train}$); $i=1, 2, \ldots, N/n$. Iterative optimization of the pre-trained model, $m \epsilon $ *Model* is carried out using ‘$n$’ for a specific count of epochs to decrease the loss, $L$ by weight updation as given in eq. (15).

\begin{equation*} L(w,X_{i})=1/n\sum \limits _{x\in X_{i},y\in Y_{i}} {l(m(x,w),y)}\tag{15}\end{equation*}

The $l$ denotes the binary cross-entropy loss function and is represented in eq. (16) and $m$ ($x$, $w$) is the mathematical function that maps a category ‘$y$’ for input feature ‘$x$’ and weight ‘$w$’.

\begin{equation*} l=-\sum \limits _{c=1}^{M} {y_{o,c}} \log (p_{o,c})\tag{16}\end{equation*}

In eq. (16), $M$ is the class number, ‘$y$’ specifies whether target ‘$c$’ is the right classification for observation ‘$o$’, and ‘$p$’ is the probability that observation ‘$o$’ belongs to target class ‘$c$’. Resolving, the equations will end up in a learned model.

In the feature extraction phase, attributes are extricated from the learned MobileNetv2 model by taking activations onto the last fully connected layer (‘*new_fc*’) of the learned model.

In the classification phase, the extracted feature vector along with the corresponding targets is used for FKNN model training. The hyper-parameters of the FKNN model are fine-tuned via PF-BAT optimization using the validation accuracy as the fitness criteria (g)

\begin{equation*} g=(Accuracy)_{_{validation}}\tag{17}\end{equation*}

The step-by-step procedure for hyper-parameter tuning is elicited in the form a simple illustrative example given as Algorithm 1.

**Table:** 

Algorithm 1 Pseudo-Code for Hyper Parameter Tuning	
S1:	Parameter Initialization of PF-BAT algorithm, Number of BATs ($N$), Generation Number, $B$, $r$, min pulse frequency, i.e., $freq_{\min } $ and max pulse frequency, i.e., $freq_{\max } $
R1:	$N = 10$; Generation Number=100; $B = 0.2$; $r = 0.4$, $freq_{\min } = 0$, $freq_{\max } = 0.5$
S2:	Random generation of BAT position that encodes the hyper-parameters ${{\boldsymbol{k}}}$ and ${{\boldsymbol{b}}}$ of FKNN. They are initialized in the range [1 10]
R2:	$\underline {\boldsymbol{Exampleoutput}}$ ${{\boldsymbol{k}}}\,\,=$ [1, 4, 3, 8, 9, 2, 7, 10, 5, 6] ${{\boldsymbol{b}}}=$[2.57, 8.43, 0.63, 7.49, 8.56, 4.62, 8.94, 0.16, 2.36, 1.46]
S2.1:	FKNN training using deep features from ‘$new\_fc$’ layer of the transfer learned model by employing rounded ${{\boldsymbol{k}}}$ and ${{\boldsymbol{b}}}$
R2.1:	$\textit{Training Phase}$
S2.2:	For the validation set, predict the class labels through trained FKNN using the following $c(x)=\arg [\max (mb_{i} (x))]_{i=1}^{2} $ where $mb_{i} (x)=\frac {\sum \limits _{j=1}^{k} {mb_{ij} \left ({{\frac {1}{\vert \vert x-x_{j} \vert \vert ^{\frac {2}{b-1}}}} }\right)}}{\sum \limits _{j=1}^{k} {\left ({{\frac {1}{\vert \vert x-x_{j} \vert \vert ^{\frac {2}{b-1}}}} }\right)}} j=1,2,\ldots, k$, and $i=1,2 \ldots \ldots$,$C$
R2.2:	$\underline {\boldsymbol{Exampleoutput}}$ ${\boldsymbol{mb}}_{ \boldsymbol {i}} = ${(1 0), (0 1), (0.003 0.997), (0.42 0.58), (0.92 0.08} ${{\boldsymbol{Lb}}}_{ \boldsymbol {i}}=$ {C, N, N, N, C}
S3:	Calculate the fitness value $g=(Accuracy)_{_{validation}} $ and chose ${\boldsymbol{pbest}}_{ \boldsymbol {j}}$ for each BAT and the ${\boldsymbol{gbest}}$ from all BATs. Initially, ${\boldsymbol{pbest}}_{ \boldsymbol {j}}$ is equal to BAT position and ${\boldsymbol{gbest}} = \text{BAT}$ position having maximum value of $g$
R3:	$\underline {\boldsymbol{Exampleoutput}}$ $g =$ [0.9835, 0.9856, 0.9877,…]$_{\mathrm {1\times 10}}$ ${\boldsymbol{pbest}} =$ {(1 2.57), (4 8.43), (3 0.63),…}$_{\mathrm {1\times 10}}$ ${\boldsymbol{gbest}} =$ {(3 0.63)}
S4:	Update the generation value
R4:	Generation Number=1
S5:	Update position for all bat’s using the formula $x_{j}^{t} =\left({1-\frac {x\ast }{x_{j}^{t-1}}}\right)\times freq_{j} \times x\ast +\left({\frac {x\ast }{x_{j}^{t-1}}}\right)\times freq_{j} \times pbest_{j} $; $j$ refers to the current BAT position
R5:	$\underline {\boldsymbol{Exampleoutput}}$ ${{\boldsymbol{x}}}\,\,=$ {(2.39 1), (6.55 2.13), (1 1),…}$_{\mathrm {1\times 10}}$
S6:	Is rand (0,1) $> {{\boldsymbol{r}}}_{ \boldsymbol {j}}$, If yes go to S 7 else select new solutions using S 8
R6:	$No/Yes$
S7:	Compute a local solution $x_{new} =x_{old} +\varepsilon B^{t}$
R7:	$\underline {\boldsymbol{Exampleoutput}}$ ${{\boldsymbol{x}}}_{ \boldsymbol {new}} =$ {(2.39 1), (3.09 1.06), (2.85 2.8),…}$_{\mathrm {1\times 10}}$
S8:	If ($rand (0,1)$ < $B_{j} \,\,or\,\,\,cfit_{j} >gfit$) (${\boldsymbol{cfit}}_{ \boldsymbol {j}}$: current fitness, $gfit:\textbf{gbest fitness}$) Accept the new solutions and Increase $r_{j}$ and decrease $B_{j} $ or take constant values of $r_{j}$ and $B_{j} $ end
R8:	$\underline {\boldsymbol{Exampleoutput}}$ ${\boldsymbol{cfit}}=$[0.9835, 0.9835, 0.9877,…]$_{\mathrm {1\times 10}}$ ${\boldsymbol {gfit}} = [0.9877]$
S9:	Determine $g=(Accuracy)_{_{validation}} $ for new solutions
R9:	$\underline {\boldsymbol{Exampleoutput}}$ ${{\boldsymbol{g}}}=$[0.9835, 0.9835, 0.9877,…]$_{\mathrm {1\times 10}}$
S10:	Update the ${\boldsymbol{pbest}}_{ \boldsymbol {j}}$ If (${\boldsymbol{cfit}}_{ \boldsymbol {j}} > {\boldsymbol{pfit}}_{ \boldsymbol {j}}$) ${\boldsymbol{pfit}}_{ \boldsymbol {j}} = {\boldsymbol{cfit}}_{ \boldsymbol {j}}$; ${\boldsymbol{pbest}}_{ \boldsymbol {j}} =$ current bat position end
R10:	$\underline {\boldsymbol{Exampleoutput}}$ ${\boldsymbol{pfit}} =$ [0.9835, 0.9856, 0.9877,…]$_{\mathrm {1\times 10}}$ ${\boldsymbol{pbest}} =$ {(2.39 1), (4 8.43), (2.85 2.8),…}$_{\mathrm {1\times 10}}$
S11:	Updation is done for all the bat’s? If yes, go to S 12 else to S 5
R11:	$Yes$
S 12:	Update the gbest value by comparing ${\boldsymbol{gfit}}$ with ${\boldsymbol{pfit}}_{ \boldsymbol {j}}$ of the entire population If (${\boldsymbol{pfit}}_{ \boldsymbol {j}} > {\boldsymbol{gfit}})$ ${\boldsymbol{gbest}} = {\boldsymbol{pbest}}_{ \boldsymbol {i}}$; end
R12:	$\underline{\boldsymbol{Example \, output}}$ ${\boldsymbol{gbest}} =[2.85 2.8]$
S:13	Check if the generation end has reached. If yes go to S 14 else go to S 4
R13:	$No$
S14:	Obtain the optimal value of hyper-parameters from ${\boldsymbol{gbest}}$ as ${\boldsymbol{k\_opt}}=$ round(${\boldsymbol{gbest}}$(1)); ${\boldsymbol{m\_opt}}={\boldsymbol{gbest}}(2)$)
R14:	$\underline{\boldsymbol{Example \, output \, at \, the \, end \, of \, the \, Generations}}$ ${\boldsymbol{k\_opt}} = 3$; ${\boldsymbol{m\_opt}}=1.0346$;
S: Specifies Step R: Specifies output	

## III. Experimental Results

### A. Experimental Settings

Table 1 represents the total number of COVID scans used in training and validation. The scans are resized to $224\times 224\times 3$, matching the dimensions of the Input layer of the learned model. The weight parameters are tuned via ‘Adam’ with the initial learning rate as 0.0006 and the transfer learned model is fitted for 40 epochs using a mini-batch size of 180. Moreover, binary cross-entropy is used as a loss function to improve the accuracy of diagnosis. The learned model is executed in MATLAB 19a and implemented on Intel Core i7-4500U CPU, 8 GB RAM, and 1.8 GHz processor. Additionally, metrics such as Recall, Precision, Accuracy, the area under the curve (AUC), and F1-Score are used for the performance evaluation of the proposed diagnosis system. They are mathematically defined as

\begin{align*} Recall=&\frac {TP}{TP+FN} \tag{18}\\ Precision=&\frac {TP}{TP+FP} \tag{19}\\ Accuracy=&\frac {TN+TP}{TP+TN+FP+FN}\tag{20}\end{align*}

F-score is defined in (21) and AUC is calculated using the technique given in [33].

\begin{equation*} F-score=\frac {(1+\beta ^{2}(Precision.Recall))}{(\beta ^{2}\times Precision+Recall)}\tag{21}\end{equation*}

In the above equation $\beta $ is taken as 1

### B. Results

**FIGURE 4.**
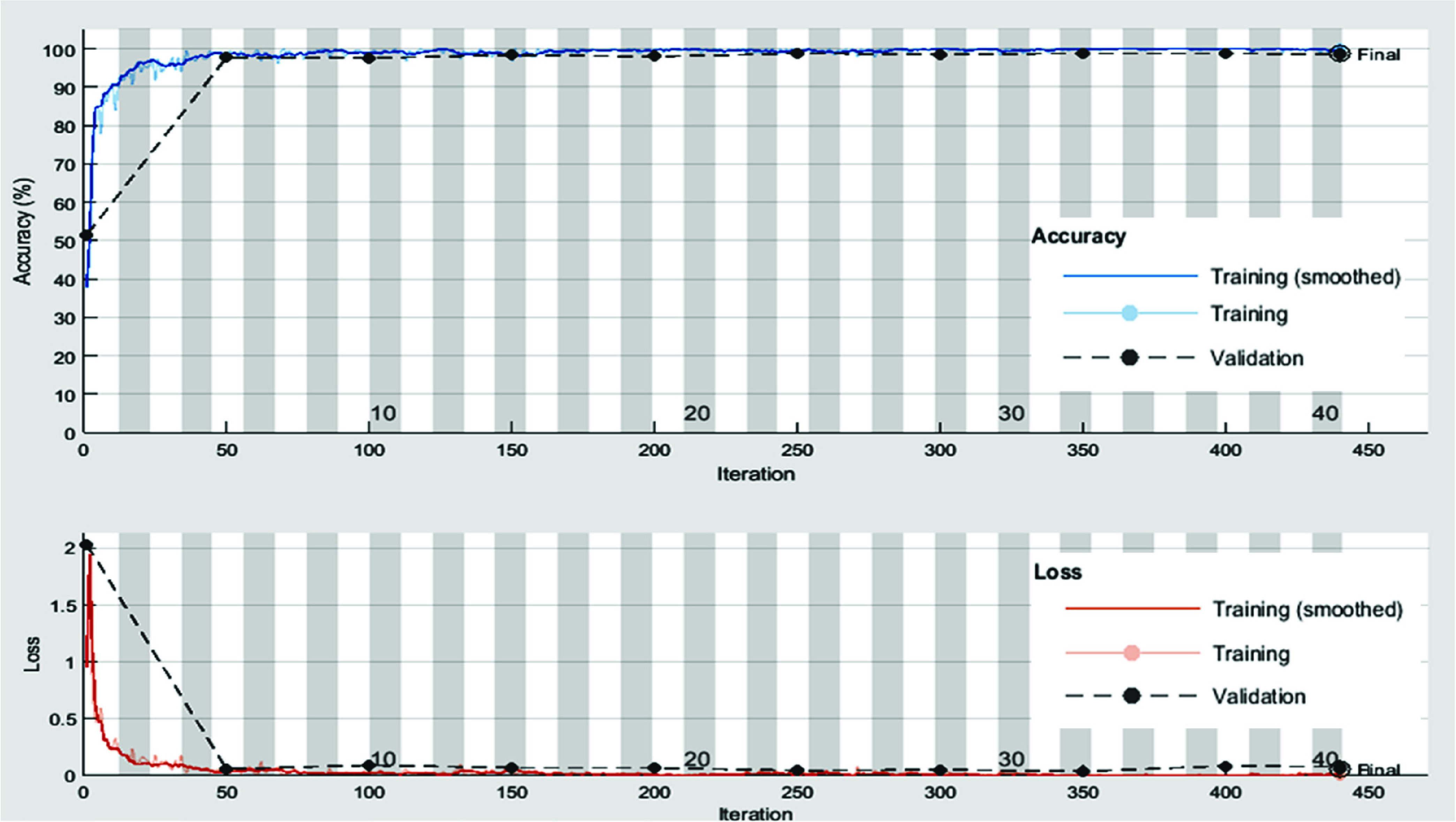
Training progress and loss curves for the transfer learned MobileNetv2 model.

We have presented the experimental results obtained using transfer learned MobileNetv2 model and proposed PF-BAT enhanced FKNN model (employing features from the ‘*new_fc*’ layer) for the classification of COVID CT images from Non-COVID images. Firstly, the MobileNetv2 is trained on the COVID CT scan image database and thereafter discriminative features are extracted from the ‘*new_fc*’ layer. The deep features from the ‘*new_fc*’ layer are used to train an FKNN whose hyper-parameters are fine-tuned using the PF-BAT. Fig. 4 shows the training progress and loss curve for the transfer learned MobileNetv2 model for 40 epochs. Table 4 gives the results for learned MobileNetv2, and proposed method. The comparative tabular values show that the proposed PF-BAT enhanced FKNN method using features from the ‘*new_fc*’ layer performs the best by achieving ceiling level validation accuracy of 99.38%, precision of 99.20%, recall of 99.60%, F1-score of 99.40%, and AUC of 99.58%.

**TABLE 4:** Performance Metrics for the Transfer Learned and Proposed Method on the Validation Data Set

Classification Algorithm	Precision	Recall	Accuracy	F1-score	AUC
Transfer Learned MobileNetv2	98.80%	98.80%	98.77%	98.80%	99.44%
Proposed Model	99.20%	99.60%	99.38%	99.40%	99.58%

**FIGURE 5.**
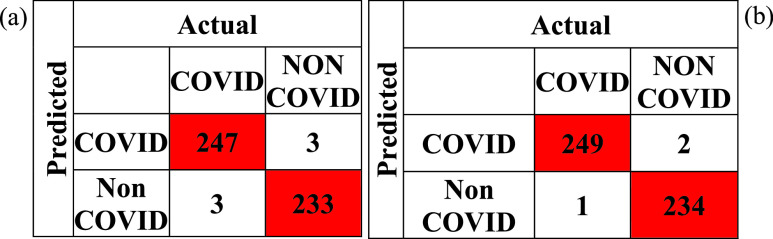
Confusion matrix for the (a) transfer Learned MobileNetv2 model, (b) proposed method.

**FIGURE 6.**
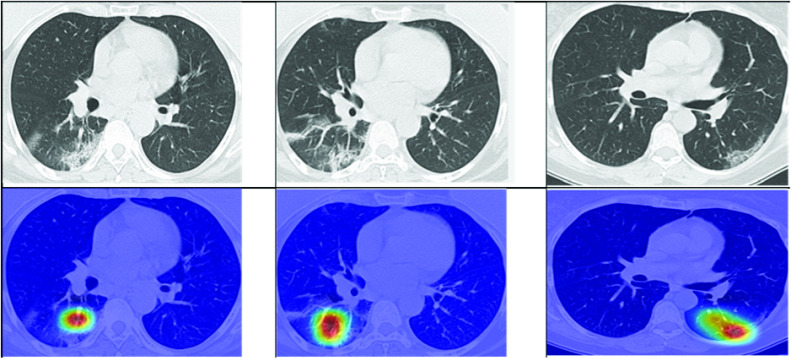
Occlusion Sensitivity visualizations for the proposed technique for COVID positive scan predictions.

Fig. 5 shows the confusion matrix for transfer learned MobileNetv2 and the proposed PF-BAT enhanced method. The FN + FP, i.e., 3, is less in the proposed method than in learned MobileNetv2, i.e., 6 resulting in small miss classification error. Fig. 6 shows the Occlusion Sensitivity visualizations for the proposed optimized FKNN classifier predictions. Visualizing the figure reveals that the proposed technique focuses on the image areas decisive for COVID-19 detection avoiding the false image edges and corners. Clearly, the activations are localized within the lungs.

**FIGURE 7.**
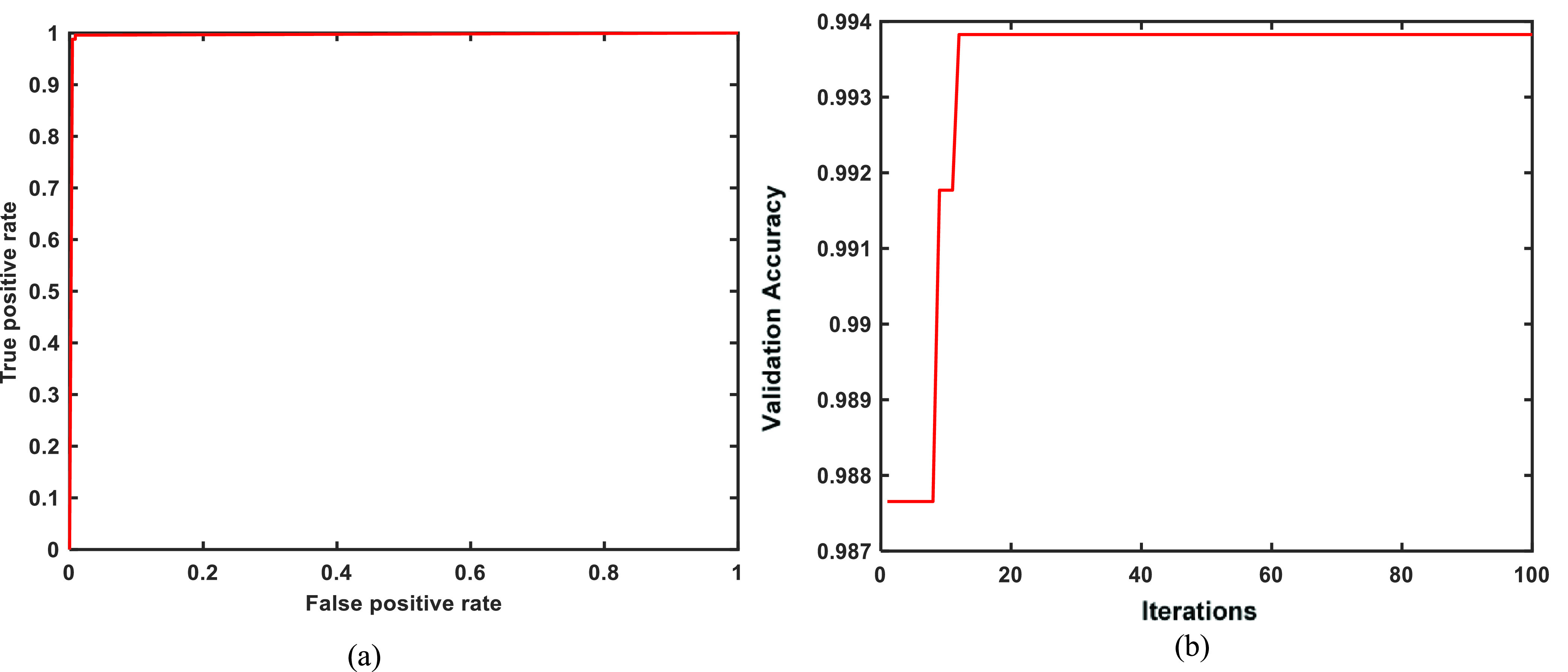
(a) AUC curve for the proposed model, (b) Fitness vs iteration curve for MobileNetv2+PF-BAT enhanced FKNN method.

Fig. 7 (a) represents the AUC curve for the proposed model indicating that the model reaches sensitivity vs 1-specificity value close to 1, i.e., 99.58%. The increase in validation performance is attributed to the PF-BAT optimized classifier, where improvised BAT is used to fine tune hyper-parameters to reduce loss. Table 5 gives the optimized value of the nearest neighbor ‘$k$’ and fuzzy strength parameter ‘$b$’ that improved the validation accuracy to 99.38%. Fig. 7(b) shows the fitness vs the generation/iteration curve. Clearly, a surge in the validation accuracy is seen using the optimal value of the hyper-parameters for the FKNN classifier, i.e., ‘${{\boldsymbol{k}}}$’ = 3, and ‘${{\boldsymbol{b}}}$’ = 1.0346. Merely, in less than 20 iterations, the accuracy improved from 98.77% to 99.38%.

**TABLE 5:** Optimal Value of the Hyper-Parameters Obtained via PF-BAT Optimization Algorithm

Hyper Parameter	Optimal Value
Nearest Neighbor (‘${k}$’)	3
Fuzzy Strength Parameter (‘${b}$’)	1.0346

## IV. Discussion

Besides exploring the efficiency of the proposed PF-BAT-based FKNN for COVID detection, a comparative assessment is conducted with the state-of-the-art methods. The comparison has been constrained to the work reported on the same database [14], [15], [34] only. As evident from Table 6, the proposed method yielded an accuracy of 99.38% and F-score 99.40% that is superior to the reported state-of-the-art techniques (accuracy of 97.38%, 97.23%, 98.37%, 98.99% and F-score of 97.31%, 97.89%, 98.14% respectively reported in literatures [14], [15], [34]). Soares *et al.* [14] have proposed $x$-DNN model for coronavirus detection, considering attributes from the ‘*fc*’ layer of VGG-16. They have also tried to improve the computational complexity with various other models like GoogleNet, Alexnet, Decision Trees, and AdaBoost classifiers. Researcher in [15] have employed the Bi-LSTM-DBM for COVID classification. In this work, MADE is used to fine tune hyper-parameters of the DBM model, where the layer number, node number, clip gradients, learning rate, number of epochs, and batch size are fine-tuned. Clearly, MADE optimization surged the classification performance than using DBM alone.

**TABLE 6:** Comparison of the Proposed Method With the Existing Works in Literature Using the Same Database Reported in [14]

Method	Accuracy	Precision	Recall	F1Score	AUC
$x$DNN [14]	97.38%	99.16%	95.53%	97.31%	97.36%
GoogleNet [14]	91.73%	90.20%	93.50%	91.82%	91.79%
VGG16 [14]	94.96%	94.02%	95.43%	94.97%	94.96%
Alexnet [14]	93.75%	94.98%	92.28%	93.61%	93.68%
Decision Tree [14]	79.44%	76.81%	83.13%	79.84%	79.51%
AdaBoost [14]	95.16%	93.63%	96.71%	95.14%	95.19%
DBM [15]	97.23%	98.14%	97.68%	97.89%	97.71%
DBM+MADE [15]	98.37%	98.74%	98.87%	98.14%	98.32%
EfficientNet [34]	98.99%	99.20%	98.80%	–	–
MobileNetv2+SVM [14]	98.35%	97.64%	99.20%	98.41%	99.12%
Proposed	99.38%	99.20%	99.60%	99.40%	99.58%
Proposed(5-Fold CV)	99.18%	98.81%	99.60%	99.20%	99.16%

Differing from the studies reported in [14] and [15], the proposed PF-BAT enhanced FKNN model offers the following advantages apart from rendering ceiling level of classification performance: 1) It eliminates the need to employ more than one pre-trained deep architectures for COVID classification, i.e., just MobileNetv2 compared to both VGG-16 and $x$-DNN used in [14], 2) The dimensionality of the optimization problem is much lower, i.e., just two compared to six reported in [15].

Apart from this, experimentations were also done in using PF-FKNN on features extracted from trained VGG16 and GoogleNet models. For VGG16, the accuracy increased to 95.47% (using optimal value of ‘${{\boldsymbol{k}}}$’ and ‘${{\boldsymbol{b}}}$’ as 9 and 1.2613) in contrast to the base value of 94.96% reported in [14]. For GoogleNet the accuracy improvised to 94.24% (using optimal value of${{\boldsymbol{k}}}'$ and ‘${{\boldsymbol{b}}}'$ as 12 and 6.1158) from the base value of 91.73%.

In order to cross validate the performance of the proposed model on another dataset, the COVID19-CT images provided by He *et al.* [11], [35] has been used in the given study. In this database, 349 + *ve* and 397 − *ve* COVID scans of different sizes are available. Training, test, and validation sets were created by dividing the data in the ratio of 0.6, 0.25, and 0.15 resulting in 191, 234; 98, 105; & 60, 58 + *ve* and − *ve* COVID scan images in the respective pools without any prior information (whether they were subject independent or not [11], [35]). We have followed the same data split ratio according to the base paper to get fair comparison with existing state of artwork. Table 7 summarizes experimental results on this dataset. The proposed model attains ceiling level of classification accuracy by achieving test accuracy and F1-score of 84.24% of 83.16% respectively. The obtained results are superior to other proposed models (e.g. CRNet [35], CVR-Net [36], MNasNet1.0 [37], and Light CNN [38], those yielded an accuracy of 73%, 78%, 81.77%, and 83% respectively).

**TABLE 7:** Comparative Performance Analysis of the Proposed Model With the Existing Techniques [35]

Method	Accuracy	F1 Score
VGG-16 [35]	76.00%	76.00%
ResNet-18 [35]	74.00%	73.00%
ResNet-50 [35]	80.00%	81.00%
DenseNet-121 [35]	79.00%	79.00%
DenseNet-169 [35]	83.00%	81.00%
EfficientNet-b0 [35]	77.00%	78.00%
EfficientNet-b1[35]	79.00%	79.00%
CRNet [35]	73.00%	76.00%
CVR-Net [36]	78.00%	78.00%
MNasNet1.0[37]	81.77%	83.56%
ShuffleNet-v2-x1.0[37]	74.38%	75.70%
Light CNN [38]	83.00%	83.33%
MobileNetv2+SVM	81.77%	79.78%
Proposed Algorithm	84.24%	83.16%
Proposed (5Fold-CV)	88.21%	87.06%

Apart from validating the performance of the proposed MobileNetv2+PF-BAT enhanced FKNN on the binary classification task (COVID Vs Non-COVID), the evaluation over larger multiclass data has also been carried out. The experimentations were done on multiclass dataset having 4173 CT scans from 210 patients [14]. The total scans were further divided into 758, 2168, and 1247 2D images with Healthy, COVID, and patients with other pulmonary conditions as labels as per the research conducted by Soares *et al* [14]. The overall test accuracy from this dataset using the proposed MobileNetv2+PF- BAT enhanced FKNN model is 89%, F1 score is 88%, Sensitivity is 89.41%, Specificity is 94.73%, Precision is 87.03%, and AUC is 96.94% under the train, test, and validation splits of 0.6, 0.2, and 0.2. Employing five-fold cross-validation strategy, the mean test accuracy of 95.99%, F1 score of 95.47%, Sensitivity of 95.54%, Specificity of 97.81%, Precision of 95.43%, and AUC of 97.77% is obtained. The validations over a large multiclass dataset also justify that the proposed approach can achieve a ceiling level of classification performance.

## V. Conclusion

In this present work, a novel model is proposed that automatically identifies the COVID + *ve* signature from the lung radiographs. In the proposed model, transfer learned MobileNetv2 is used as a feature extractor. The discriminative features extracted from the fully connected layer of the learned model are fed to the PF-BAT enhanced FKNN classifier. The hyperparameters of the FKNN have been optimized using the PF-BAT algorithm. Thereafter, the proposed model has been extensively validated on publicly available CT scan image datasets. The analysis on the datasets reveals that with the scheme of hyper-parameter optimization, an increase in the validation accuracy is obtained. The accuracy improves by 0.617%, 1.189%, and 3.851% respectively with optimization. The comparative analysis shows that the proposed model outperforms the existing state-of-the-art models. Consequently, the proposed model can work as a fast automated intelligent tool for assisting healthcare professionals in decision making.
